# Diffusion tensor imaging in neuropsychiatric systemic lupus erythematosus

**DOI:** 10.1186/1471-2377-10-65

**Published:** 2010-07-28

**Authors:** Rex E Jung, Arvind Caprihan, Robert S Chavez, Ranee A Flores, Janeen Sharrar, Clifford R Qualls, Wilmer Sibbitt, Carlos A Roldan

**Affiliations:** 1 The Mind Research Network, Domenici Hall, 1101 Yale NE, Albuquerque, New Mexico, 87106, USA; 2 Department of Neurosurgery, University of New Mexico Health Sciences Center, 1 University of New Mexico, Albuquerque, New Mexico, 87131, USA; 3 Department of Internal Medicine, University of New Mexico Health Sciences Center, 1 University of New Mexico, Albuquerque, New Mexico, 87131, USA; 4 Department of Mathematics and Statistics, University of New Mexico, 1 University of New Mexico, Albuquerque, New Mexico, 87131, USA

## Abstract

**Background:**

Neuropsychiatric systemic lupus erythematosus (NPSLE) is associated with increased morbidity and mortality.

**Methods:**

We used Diffusion Tensor Imaging (DTI) to assess white matter abnormalities in seventeen NPSLE patients, sixteen SLE patients without NPSLE, and twenty age- and gender-matched controls.

**Results:**

NPSLE patients differed significantly from SLE and control patients in white matter integrity of the body of the corpus callosum, the left arm of the forceps major and the left anterior corona radiata.

**Conclusions:**

Several possible mechanisms of white matter injury are explored, including vascular injury, medication effects, and platelet or fibrin macro- or microembolism from Libman-Sacks endocarditis.

## Background

Neuropsychiatric systemic lupus erythematosus (NPSLE) is a complex neurological disorder characterized by neuropsychological dysfunction [1]. Patients can present with either focal symptoms, consisting of stroke and/or transient ischemic attacks, or with central nonfocal symptoms of cognitive dysfunction, acute confusional state, seizures, or psychosis. NPSLE is likely initiated by inflammatory, thrombotic, and/or cardioembolic etiologies and further exacerbated by antibody, cytokine, and cytotoxin mediators [2,3]. Neuropsychiatric manifestations, affecting some 75% of patients diagnosed with systemic lupus erythematosus (SLE), are associated with significantly increased morbidity and mortality [4]. Indeed, previous studies have demonstrated a wide range of brain abnormalities in NPSLE both during and after experiencing acute symptoms [5-8].

Diffusion Tensor Imaging (DTI) offers increased resolution compared to conventional structural Magnetic Resonance Imaging (sMRI) regarding white matter microstructure by measurement of water diffusion through cellular compartments *in vivo *[9]. Compared to more isotropic movement of water in gray matter, water diffusion in white matter moves anisotropically, meaning that water diffuses preferentially along the length of the axon compared to perpendicular to the axon. This anisotropic diffusion of water appears to be due to the highly structured axonal membranes and their associated myelin sheaths [10]. By tracking the diffusion of water in the brain, the measure *fractional anisotropy *(FA) and *mean diffusivity *(MD) can be derived. Higher FA (and lower MD) suggests greater axonal coherence and myelination [9], increasing in a roughly linear manner that conforms to normal developmental brain processes [11,12]. Measures of FA are usually considered to be overall measures of axonal integrity, reflecting either increased axonal caliber, increased myelin thickness, increased fiber coherence in a given direction, or some combination of these factors [13]. In contrast, MD, is a measure of the average molecular motion independent of the constraints of tissue boundaries, and is affected by cellular size and degradations in tissue integrity [14].

Relatively few studies have emerged showing water diffusivity changes in NPSLE. The first study compared 9 active to 10 chronic NPSLE patients using magnetization transfer imaging (MTI), a technique designed to compare bound to free protons in biologic tissue [15]. These researchers found that MTI values in active NPSLE patients differed significantly from chronic patients, who were similar to controls, interpreted to reflect the presence of inflammation in the active cohort. A second group used a region of interest (ROI) approach to assess a broad cohort of 34 patients diagnosed with SLE, using diffusion weighted imaging, a technique sensitive to the microscopic motion of water within extracellular space [16]. They found early diffusion changes in the frontal lobe, the genu of the corpus callosum, and the anterior internal capsule, even in the presence of normal magnetic resonance imaging (MRI) findings. Finally, a group compared 8 female NPSLE patients, with new onset of symptoms, to 20 healthy controls using diffusion tensor imaging (DTI). Using an ROI approach, they found that these patients differed from controls in a wide range of normal appearing gray and white matter regions including the insula, thalamus, parietal and frontal white matter, and corpus callosum [17].

Tract Based Spatial Statistics (TBSS) is an automated analysis methodology allowing for projection of individual subject diffusion tensor maps into a common space, allowing for localized statistical testing across the entire brain as opposed to ROI approaches [18]. TBSS is a relatively new analysis technique that has been applied to a broad range of neurological and psychiatric disorders to assess the microstructural integrity of white matter [18-20]. We sought to use DTI and automated TBSS to assess WM abnormalities in a cohort of patients comprised of acute NPSLE, SLE without NPSLE, and normal controls. We hypothesized greater WM abnormalities in NPSLE compared to either SLE or controls.

## Methods

### Sample

The sample consisted of thirty-three SLE patients, recruited from the Rheumatology Clinics of the University of New Mexico, ranging in age from 18 to 60 (94% female). All subjects were diagnosed with SLE based on the 1997 update to the 1982 American College of Rheumatology Revised Criteria for Classification of Systemic Lupus Erythematosus [21]. Seventeen of the SLE patients had acute NPSLE defined as acute stroke or transient ischemic attack (TIA), acute confusional state, moderate cognitive dysfunction, seizures, or psychosis. Sixteen patients had SLE, but no past or acute NPSLE.

These 2 groups of patients were compared to twenty healthy controls. All participants signed a consent form approved by the institutional review board of the University of New Mexico, and consistent with the Declaration of Helinski, prior to participation in the experimental protocol. Participants were screened for conditions that would prohibit undergoing an MRI scan (e.g., metal implant, orthodontic braces, severe claustrophobia).

### Clinical Measures

The Systemic Lupus Erythematosus Disease Activity Index (SLEDAI) [22] and Systematic Lupus International Collaborating Clinics/America College of Rheumatology Damage Index (SLICC/ACR DI) [23] were administered by an experienced rheumatologist (WLS) to each patient. Clinical diagnosis of NPSLE was defined by presence of past or current: stroke, transient ischemic accident, psychosis, seizure disorder, confusional state, and/or moderate or severe cognitive dysfunction. No SLE patients had any past or current evidence of any of these clinical diagnoses. Table 1 summarizes the clinical characteristics of the NPSLE subjects.

**Table 1:** NPSLE - Clinical Characteristics

Subject	Group	New Stroke	New TIA*	Acute Seizure	Acute Cognitive Decline	Acute Confusional State	New Psychosis
1	NPSLE		Yes				
2	NPSLE	Yes	Yes		Yes	Yes	Yes
3	NPSLE		Yes				
4	NPSLE		Yes				
5	NPSLE		Yes				
6	NPSLE			Yes			
7	NPSLE		Yes				
8	NPSLE				Yes		
9	NPSLE		Yes				
10	NPSLE		Yes				
11	NPSLE	Yes		Yes	Yes	Yes	
12	NPSLE		Yes				
13	NPSLE		Yes				
14	NPSLE	Yes					
15	NPSLE	Yes	Yes				
16	NPSLE	Yes	Yes		Yes	Yes	
17	NPSLE	Yes	Yes		Yes	Yes	

Clinical Characteristics of NPSLE patient cohort. Six patients had new strokes, thirteen had new transient ischemic attacks, two presented with acute seizures, two presented with acute cognitive deficits, four presented with acute confusional states, and one presented with new onset psychosis. These symptoms are consistent with a diagnosis of NPSLE.

*TIA = Transient Ischemic Attack

### Behavioral Measure

The Wide Range Achievement Test - 3^rd ^Revision (WRAT-3) Reading subtest, was used as a measure of premorbid cognitive functioning. This measure requires subjects to read single words with irregular phonetic spelling (e.g., colonel), and has been found to be resistant to the effects of cognitive decline due to neurological or psychiatric disease [24].

### Radiological Read

All structural scans were read by a neuroradiologist, blinded to group status. Classifications were made on presence and location of cortical atrophy, white matter lesions, and old or recent infarcts. Table 2 summarizes significant radiological abnormalities found for NPSLE and SLE subjects. Fisher's exact tests were performed to assess group differences across domains.

**Table 2:** Radiological abnormalities in NPSLE and SLE patients.

Subject	Group	Atrophy	Subcortical White Matter	Periventricular White Matter	Deep White Matter	Old Infarct	Recent Infarct
1	NPSLE			Yes	Yes	R Frontal	

2	NPSLE			Yes	Yes		

3	NPSLE		General	Yes		L Temporal/ Occipital	

4	NPSLE						

5	NPSLE						

6	NPSLE						

7	NPSLE	General		Yes	Yes	R Frontal	

8	NPSLE						

9	NPSLE	General	General	Yes		R Frontal; Cerebellum	

10	NPSLE			Yes			

11	NPSLE	General		Yes			

12	NPSLE						

13	NPSLE						

14	NPSLE		R/L frontal/ parietal	Yes	Yes		

15	NPSLE		R/L frontal	Yes		Thalamus	

16	NPSLE						

17	NPSLE			Yes	Yes	R Occipital	R Occipital

18	SLE		R frontal	Yes	Yes		

19	SLE						

20	SLE		R frontal				

21	SLE		R/L frontal; L parietal	Yes			

22	SLE		R/L frontal				

23	SLE						

24	SLE		R/L frontal; L parietal				

25	SLE		R/L frontal	Yes			

26	SLE						

27	SLE						

28	SLE		R frontal				

29	SLE		R/L frontal; R/L parietal				

30	SLE						

31	SLE		L frontal				

32	SLE		R/L frontal	Yes	Yes		

33	SLE						

Radiological findings for patients diagnosed with NPSLE and SLE. Three NPSLE patients had cortical atrophy. Four NPSLE and ten SLE patients had subcortical white matter lesions. Ten NPSLE and four SLE patients had periventricular white matter lesions. Five NPSLE and two SLE patients had deep white matter lesions. Six NPSLE patient had old infarcts. One NPSLE patient had recent infarct. NPSLE did not differ significantly from SLE patients on radiological reads except for old infarcts (Fisher's exact = .0184).

R = Right Hemisphere; L = Left Hemisphere; R/L = Right and Left Hemisphere

### Image Acquisition

MR examinations were performed on a 1.5T Siemens Sonata scanner using an 8-channel phased array head coil. Subjects' heads were stabilized with tape across the forehead and padding around the sides. Diffusion Tensor Imaging (DTI) was used to assess white matter integrity in NPSLE, SLE, and healthy subjects. We employed a single shot EPI sequence. The DTI data was collected along the anterior commissure/posterior commissure line, with FOV = 256 × 256 mm, 128 × 128 matrix, slice thickness of 2 mm (isotropic 2 mm resolution), NEX = 2, TE = 92 ms, TR = 10000 ms. We used 12 gradient directions with b = 1000 s/mm^2^. The total acquisition time was 4.32 minutes. The DTI experiment was repeated twice to increase signal-to-noise ratio.

### Data Processing

The majority of the processing was done in FSL 4.1 http://www.fmrib.ox.ac.uk/fsl.

#### Conversion to nifti

The dicom files were converted to nifti using the dicom2nii program http://www.sph.sc.edu/comd/rorden/dicom.html. This program also outputs the gradient direction tables after correction for image slice orientation and a b-value table. The two DTI experiments were concatenated into one 4 D nifti file and a concatenated table of corresponding b-value and gradient direction tables were also concatenated.

#### Eddy current correction

Eddy current correction consists of registering all the images to a b = 0 s/mm^2 ^diffusion image. We used FLIRT (FSL) with a mutual-information cost function for this step. The algorithm registers images of both the DTI measurements to a common image. The data is not averaged for the next step.

#### Calculation of diffusion tensor

The diffusion tensor, scalar diffusion parameters (MD, AD, RD, and FA) were calculated by DTIFIT (FSL).

#### Image registration for group analysis

The fractional anisotropy (FA) image of each subject was normalized to a 1 × 1 × 1 mm^3 ^FA template (FMRIB58_FA_1mm) in the Montreal Neurological Institute (MNI) space using the non-linear registration algorithm FNIRT (FSL). The spatial normalization transformation obtained by registering FA was then applied to other diffusion images (MD, AD, RD).

#### Image skeletons for group analysis

A mean FA image was calculated from the mean FA images of individual subjects. The white matter regions for this mean image were skeletonized using TBSS Version 1.1 (FSL) [18]. A threshold of FA > 0.2 defined the white matter regions. Values of FA of each subject were then projected onto the common skeleton (TBSS). The standard TBSS algorithm in FSL was used for this purpose. It consists of doing a search in the direction perpendicular to the skeleton and assigning the maximum value of FA to the skeleton. The spatial coordinate of this maximum FA value is noted and skeletons of MD, RD, and AD images are calculated by assigning the corresponding diffusivity values to the skeleton. At the end of this step we have skeleton images corresponding to FA, MD, RD, and AD for each subject. The spatial map of the skeleton is the same for each subject but the values it takes is subject dependent. All processing is done by standard TBSS algorithms and further explanation of TBSS is described in [18].

#### Statistical group analysis

We assessed group FA differences using FSL's General Linear Model (GLM) tool. Age and sex were entered into the model as nuisance variables. We thresholded the t-statistic images at t > 3.0 as recommended by FSL. The group mean differences (two-tailed) were tested using permutation methods with FSL's Randomize. We ran 5000 two-tailed Monte Carlo permutation tests for each of the group differences. All presented results are corrected p-values at p < .05 after controlling for family wise error rate. Next, we created a mask image for significant FA clusters by binarizing the FA image for results that were significant at p < .05. Our approach was to first compare SLE patients to controls, then acute NPSLE patients to controls, and finally acute NPSLE patients to SLE patients. Significant clusters were dilated for figure presentation.

## Results

The seventeen NPSLE (Mean Age = 38.6 +/- 11.8; 94% Female; WRAT-Reading = 44.3 +/- 7.7) and sixteen SLE with no NPSLE (Mean Age = 37.4 +/- 13.2; 94% Female; WRAT-Reading = 44.4 +/- 8.5) were compared to the twenty healthy controls (Mean Age = 32.5 +/- 10.7; 90% Female; WRAT-Reading = 47.5 +/- 7.3) across major demographic variables. The three groups did not differ significantly from each other in terms of age (F = 1.44, p = .25), gender (Chi-Square = .28, Probability = .87), ethnicity (Fishers Exact p = .73), or premorbid cognitive functioning (F = .96, p = .39).

**Figure 1:**
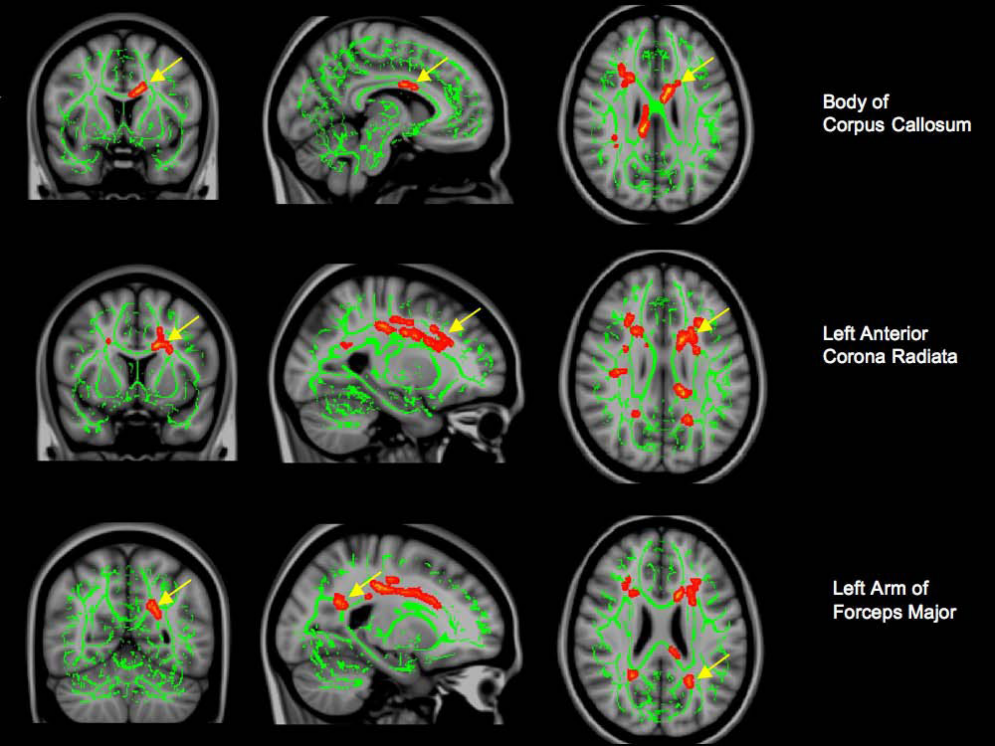
**DTI differences between NPSLE, SLE, and controls**. A) Significant regions (red/yellow) in which acute NPSLE patients had significantly lower FA than controls. B/C) Significant regions (red/yellow) in which acute NPSLE patients had significantly higher MD than controls. Yellow arrows indicate regions in which NPSLE patients had significantly lower FA than both SLE patients without NPSLE and controls. Left - coronal view; middle - sagittal view; right - axial view. Green represents the center of major white matter tracts.

Next, we sought to compare the groups across DTI measures of FA and MD. SLE patients did not differ from control subjects on either FA or MD measures after controlling for age and sex (df = 32). When NPSLE subjects were compared to control subjects, numerous regions of significant FA and MD differences were observed (Figure 1a/b).

Significant regions of differences between acute NPSLE patients and controls, controlling for age and sex (df = 33), are presented in Table 3.

**Table 3:** DTI differences between NPSLE patients and controls

FA Controls > NPSLE						
**Voxels**	**p-value**	**Max-t**	**MNI X**	**MNI Y**	**MNI Z**	**Approximate white matter tract**
266	0.001	3.135	30	17	18	Right superior longitudinal fasciculus
223	0.001	3.242	11	-34	25	Splenium of CC
172	0.003	3.408	-8	5	25	Body of CC
165	0.003	3.028	-26	28	12	Left anterior corona radiata
120	0.006	3.006	34	-41	28	Right superior longitudinal fasciculus
						
**MD NPSLE > Controls**						
**Voxels**	**p-value**	**Max-t**	**MNI X**	**MNI Y**	**MNI Z**	**Approximate white matter tract**
454	< 0.001	3.201	-35	-28	30	Left superior longitudinal fasciculus
426	< 0.001	3.028	-24	15	19	Left anterior corona radiata
214	0.001	4.027	22	-19	37	Right superior corona radiata
193	0.001	3.195	30	0	28	Right superior longitudinal fasciculus
130	0.002	3.624	27	-42	33	Right superior longitudinal fasciculus
119	0.002	4.09	-2	-9	10	Left anterior thalamic radiation
103	0.003	3.738	-23	-56	21	Left arm of forceps major
100	0.003	3.074	-28	24	21	Left anterior thalamic radiation
97	0.003	4.055	29	20	18	Right inferior fronto-occipital fasciculus
88	0.004	3.867	25	-53	18	Splenium of CC
73	0.007	3.167	-8	-29	24	Body of CC
64	0.009	3.128	33	-17	26	Right superior longitudinal fasciculus

DTI differences between NPSLE patients and controls. Upper section lists significant regions in which controls had greater fractional anisotropy (FA) than NPSLE patients. Lower panel lists regions in which NPSLE patients had greater mean diffusivity (MD) than control subjects. Voxels = significant contiguous voxel cluster. Max-t = t statistic of voxel cluster difference between controls and NPSLE. MNI X, Y, Z = Montreal Neurological Institute coordinates of centroid location of significant voxel cluster. CC = corpus callosum.

Finally, when comparing NPSLE to SLE patients, controlling for age and sex (df = 29), numerous regions of significant FA and MD differences were again observed. These are presented in Table 4.

**Table 4:** DTI differences between SLE and NPSLE patients

FA SLE > NPSLE						
Voxels	p-value	Max-t	MNI X	MNI Y	MNI Z	Approximate white matter tract
155	0.003	3.286	-35	-42	22	Left superior longitudinal fasciculus
113	0.008	3.153	-12	6	27	Body of CC
						
**MD NPSLE > SLE**						
**Voxels**	**p-value**	**Max-t**	**MNI X**	**MNI Y**	**MNI Z**	**Approximate white matter tract**
109	0.001	3.344	-15	-15	21	Genu of CC
73	0.006	4.107	-23	-56	22	Left arm of forceps major
70	0.006	3.052	-23	22	20	Left anterior corona radiata

DTI differences between SLE patients and NPSLE patients. Upper section lists significant regions in which SLE patients had greater fractional anisotropy (FA) than NPSLE patients. Lower panel lists regions in which NPSLE patients had greater mean diffusivity (MD) than SLE patients. Voxels = significant contiguous voxel cluster. Max-t = t statistic of voxel cluster difference between controls and NPSLE. MNI X, Y, Z = Montreal Neurological Institute coordinates of centroid location of significant voxel cluster. CC = corpus callosum.

There was one interesting regional similarity wherein NPSLE patients differed in terms of FA measures both from SLE patients and control subjects: the body of the corpus callosum [MNI (x, y, z) = (-8, 5, 25) (-12, 6, 27)]. Similarly NPSLE patients differed from both controls and SLE patients in MD measures obtained within the left arm of the forceps major [MNI (x, y, z) = (-23, -56, 21) (-23, -56, 22)], and the left anterior corona radiata [MNI (x, y, z) = (-24, 15, 19) (-23, 22, 20)].

Finally, we conducted post hoc analyses designed to determine whether the significant MD differences were being driven by the principal eigenvector (AD) or the average of the secondary eigenvectors (RD), which have been ascribed to predominantly axonal versus myelin processes respectively [25]. These post hoc analyses (Additional file 1: Supplemental Table S1) found significant AD and RD differences in all regions identified to differentiate NPSLE patients from SLE and controls including: the body of the corpus callosum [AD NPSLE > Controls MNI (x, y, z) = (-13, 15, 23); RD NPSLE > Controls MNI (x, y, z = (-13, 7, 27), RD NPSLE > SLE MNI (x, y, z) = (-14, 8, 28)], left arm of the forceps major [RD NPSLE > Controls MNI (x, y, z = (-23, -56, 21), RD NPSLE > SLE MNI (x, y, z) = (-23, -56, 22)], and left anterior corona radiata [AD NPSLE > SLE MNI (x, y, z) = (-22, 15, 25), RD NPSLE > Controls MNI (x, y, z = (-24, 15, 19).

## Discussion

The current results were obtained in a relatively young (less than 60 years old), large cohort of both acute NPSLE and SLE patients without NPSLE studied with voxel-based DTI techniques. Interestingly, there were no significant FA or MD differences observed between the sixteen SLE patients without NPSLE and the twenty matched controls, many of the SLE patients of whom had subcortical white matter and periventricular lesions. This could be due to the lower disease burden of this cohort, which did not reach a "threshold" detectable by our imaging and statistical methodology, or due to the non-overlapping lesion burden of the SLE cohort as compared to the NPSLE cohort. It will be important to determine in future studies, with larger samples, whether there are systematic effects of NPSLE that accumulate in the brain in such a way that they affect white matter in a systematic (as opposed to sporadic) manner, as this preliminary study suggests.

In contrast, when comparing the acute NPSLE patients to controls, we found numerous FA and MD changes reflecting diffuse white matter abnormalities. These abnormalities were also present when comparing acute NPSLE to SLE patients, suggesting that either the acute effects of the NPSLE disease, or its treatment, results in white matter changes discernable with conventional MRI techniques. Moreover, in post hoc analyses, we were not able to differentiate AD or RD changes as predominant in driving MD changes. Thus, we are unable to definitively say whether axonal or myelin processes drive the increase in MD; rather, the increased MD in the white matter regions appears to reflect increased overall diffusivity both along and perpendicular to the axon. Again, future longitudinal studies, at various stages in the acute symptomatology will be necessary to determine how disease treatment and symptom resolution are reflected in white matter changes reflected in FA and MD signal changes.

Several possible mechanisms could explain diffusion differences affecting acute NPSLE patients. The first includes immune-mediated vascular or neuronal injury and subsequent neuronal and metabolic dysfunction resulting in edematous processes that increase water content in WM regions of the brain [26]. The second relates to therapy, whereby the introduction of corticosteroids, or other immunosuppression drugs (i.e. cyclophosphamide), and/or disease modifying antirheumatic drugs, can affect water content in the brain parenchyma. The third mechanism may be related to platelet or fibrin macro- or microembolism from Libman-Sacks endocarditis or anticardiolipin antibodies causing multiple areas of macroscopic or microscopic ischemia, infarctions, and microhemorrhages with surrounding edema [2]. Decreased FA, in the same subject, is harder to interpret, being potentially related to demyelination, axonal loss, ischemia, and/or inflammation [27]. However, metabolic changes, observed with proton magnetic resonance spectroscopy, have been found in acute NPSLE, suggesting demyelination, reactive brain inflammation, ischemia or infarction as likely mechanisms leading to decreased FA values [8].

The regions where lower FA and higher MD were found in acute NPSLE are not where patients generally accumulated lesions related to large infarcts or chronic ischemic damage, although diffuse microscopic ischemic injury not detected on MRI is common on histopathologic studies of NPSLE. It is of note that none of these regions were identified on radiological scan to be regions of old infarct in NPSLE (Additional file 2: Supplemental Images S2). Thus, these results likely reflect acute injury effects of NPSLE as opposed to cumulative disease processes. One previous study of eight acute NPSLE patients (6 treated with steroids), found decreased FA in the thalamus, corpus callosum and parietal and frontal WM [17]. A second study of thirty-four patients with SLE, found diffusion abnormalities limited to the corpus callosum when patients were compared to controls [16]. The current results provide new evidence that FA and MD may have diagnostic use in NPSLE by demonstrating, for the first time, regional brain specificity, and by distinguishing NPSLE from SLE patients, further indicating the potential diagnostic specificity of this technique for patients in the acute stage of this difficult disease.

Strengths of the current study include: 1) the relatively large patient cohort, 2) the relatively youth (less than 60 years old) of the patient cohort compared to previous studies, 3) whole brain as compared to ROI analyses, 4) ease and reproducibility of TBSS methodology, and 5) the lack of differences between SLE patients without NPSLE and controls strengthen the specificity of our findings. A limitation of the study is the lack of repeated measures of the acute NPSLE patients as they progress through the acute phase of their disease. This would help to establish whether therapy or disease characteristics predominated over time in determining DTI abnormalities. However, these extremely ill patients are difficult to study repetitively in the acute setting, and these data would have likely increased the differences between acute NPSLE as compared to SLE patients or healthy controls. Future studies will determine if patients can be subcategorized into more tractable groups amenable to sensitive neuroimaging studies.

These results suggest that great care is needed when selecting NPSLE patients to participate in neuroimaging studies. Patients with SLE but no NPSLE appear to have different diffusion characteristics than those with acute symptoms (e.g., seizure, transient ischemic attack, acute confusion). These differences, in turn, appear to affect significantly the diffusion parameters in multiple white matter regions throughout the brain. As water diffusivity is critically important to the interpretation of numerous imaging paradigms, including functional magnetic resonance imaging (fMRI), perfusion weighted imaging (PWI), and magnetic resonance spectroscopy (MRS), the current data would suggest that these patients be treated as a different cohort with respect to imaging analyses.

## Conclusions

We found significant FA and MD changes in the white matter of acute patients diagnosed with NPSLE. SLE patients were not significantly different from control subjects on FA or MD measures. These results reflect an automated, replicable, and sensitive assay of white matter abnormalities in the acute phase of neuropsychiatric lupus, requiring less than 10 minutes of imaging on a standard MRI system.

## Competing interests

The authors declare that they have no competing interests.

## Authors' contributions

REJ participated in the design of the study and drafted the manuscript. AC conducted and oversaw DTI analysis. RSC conducted DTI analysis. RAF conducted cognitive evaluations and database management. JS conducted subject recruitment, qualification, retention, and care. CRQ participated in the design of the study, conducted statistical analyses, and drafted the manuscript. WS participated in the design of the study and drafted the manuscript. CAR participated in the design of the study and drafted the manuscript. All authors read and approved the final manuscript.

## Pre-publication history

The pre-publication history for this paper can be accessed here:

http://www.biomedcentral.com/1471-2377/10/65/prepub

## Supplementary Material

Additional File 1 **Post Hoc analyses comparing NPSLE, SLE, and Control subjects on measures of axial diffusivity (AD) and radial diffusivity (RD)**. contains excel file showing regions in which NPSLE patients differed significantly from SLE and Control subjects with respect to AD and RD. Click here for file

Additional File 2 **Proton Density images showing regions of white matter hyperintensities in seventeen NPSLE subjects**. contains pdf file of axial Proton Density images, for 17 NPSLE subjects, obtained at the upper margin of the lateral ventricles. Click here for file
