# Supplementary figures and images for: Diffusion tensor imaging in neuropsychiatric systemic lupus erythematosus
Source: BMC Neurol. 2010 Jul 28;10:65. doi: 10.1186/1471-2377-10-65 (PMC2919505; doi:10.1186/1471-2377-10-65)

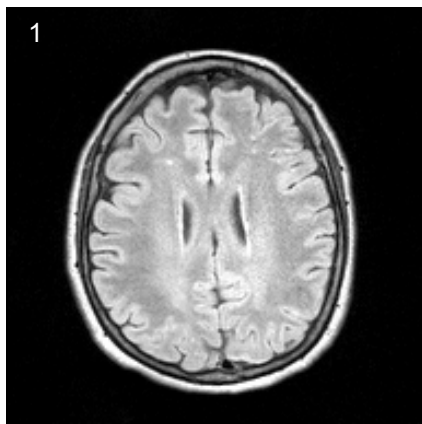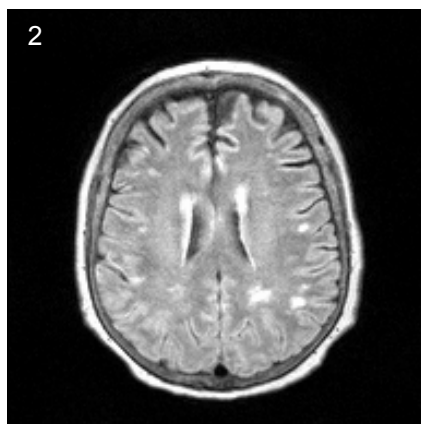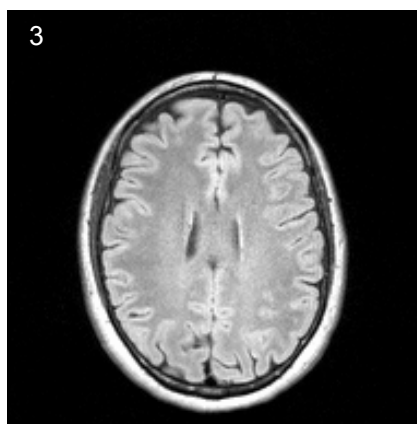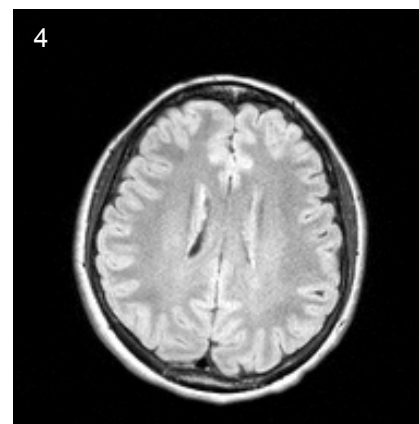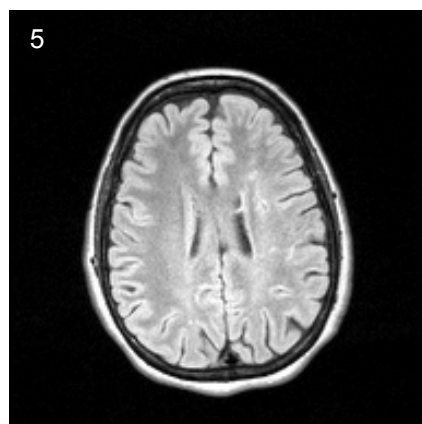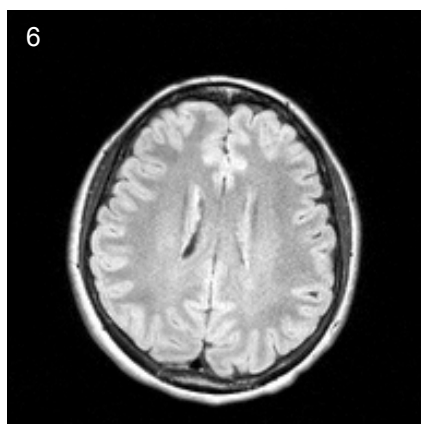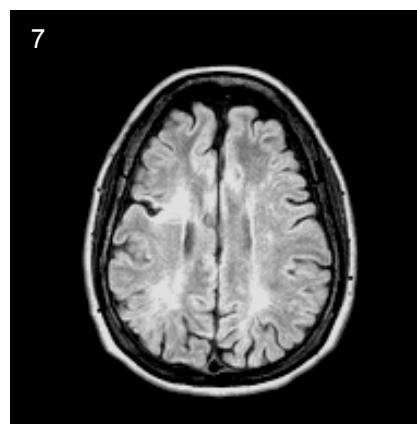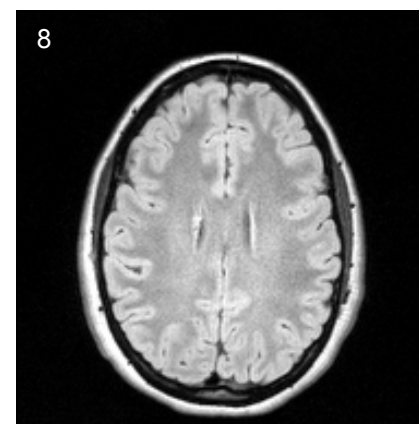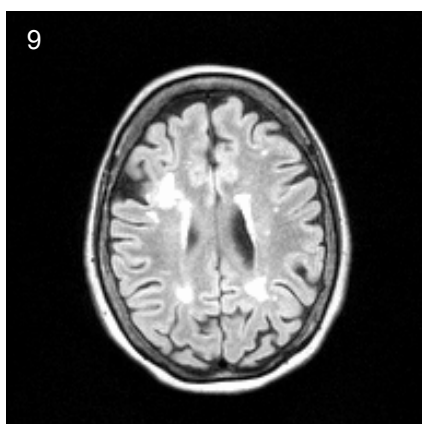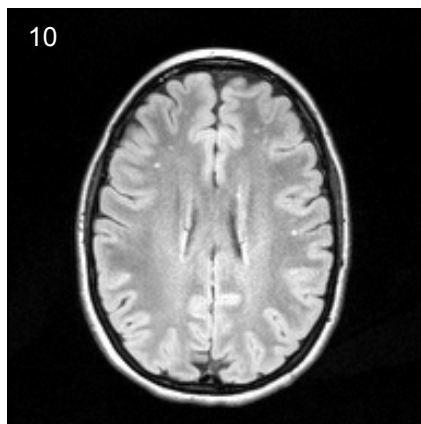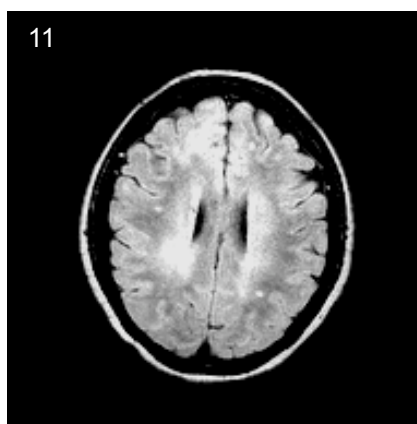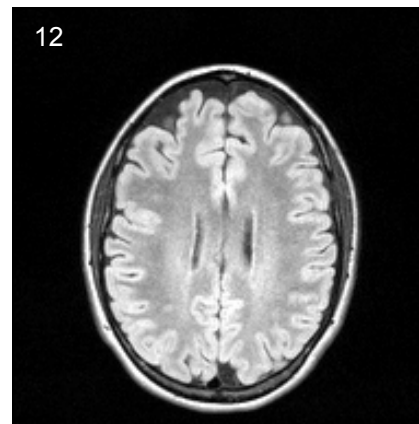

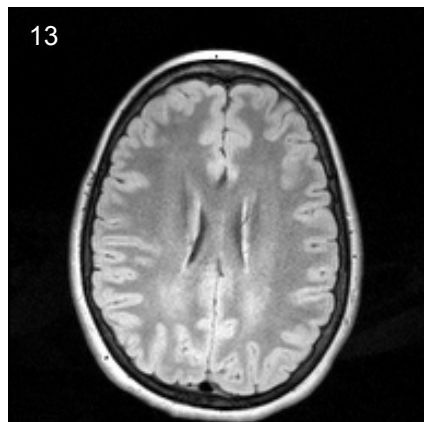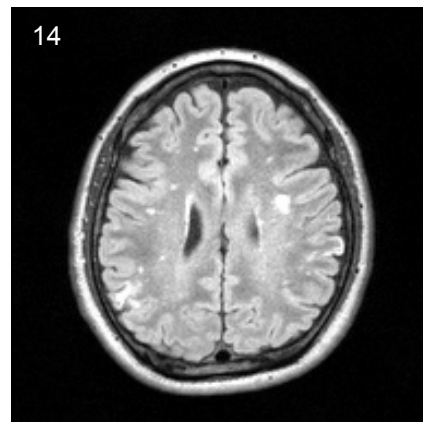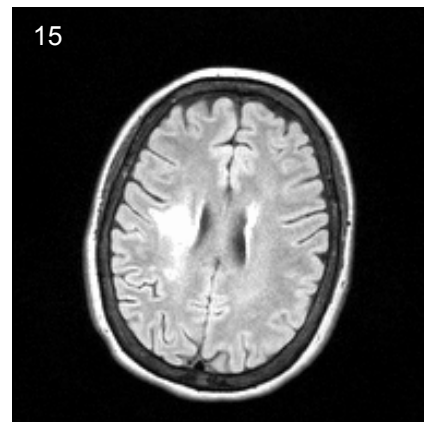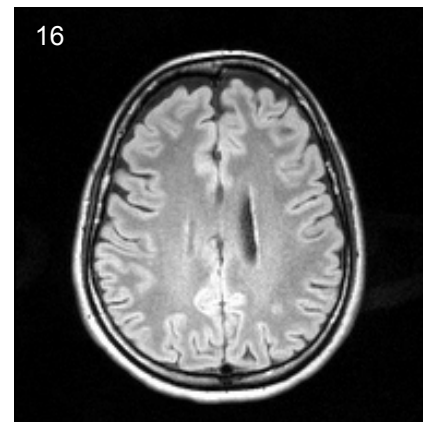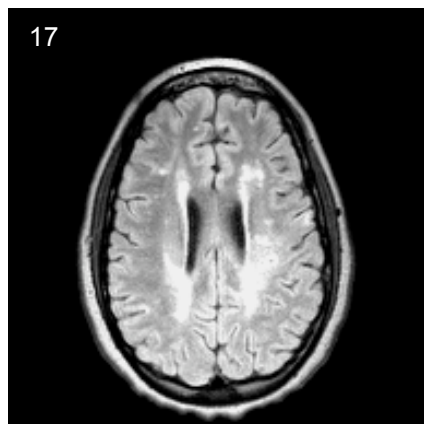

Supplement: Additional File 2 — Proton Density images showing regions of white matter hyperintensities in seventeen NPSLE subjects. contains pdf file of axial Proton Density images, for 17 NPSLE subjects, obtained at the upper margin of the lateral ventricles. [file 1471-2377-10-65-S2.PDF]
